# Acidic-responsive nano liposomes potentiate in situ dual immunotherapy on lung cancer by codelivering antagonistic peptide and agonist

**DOI:** 10.1016/j.ijpx.2026.100562

**Published:** 2026-05-06

**Authors:** Yinshan Lin, Lu Liang, Le Wang, Minyan Wei, Qingjun Yang, Xiaoling Guan, Qiuyun Liu, Youguang Pan, Jianfen Su, Lingmin Zhang

**Affiliations:** a The Affiliated Panyu Central Hospital, Guangzhou Municipal and Guangdong Provincial Key Laboratory of Molecular Target & Clinical Pharmacology, the NMPA and State Key Laboratory of Respiratory Disease, Guangdong Basic Research Center of Excellence for Respiratory Medicine, School of Pharmaceutical Sciences, Guangzhou Medical University, Guangzhou 511436, China; b Academician Workstation, Department of Pharmacy, The Jiangxi Province Key Laboratory for Diagnosis, Treatment, and Rehabilitation of cancer in Chinese Medicine, Jiangxi University of Chinese Medicine, No. 1688 Meiling Avenue, Xinjian District, Nanchang, Jiangxi 330004, China; c Department of Thoracic Surgery, Guangdong Provincial People's Hospital (Guangdong Academy of Medical Sciences), Southern Medical University, Guangzhou 510080, China

**Keywords:** Antagonistic peptides, Acidic-responsive liposomes, Tumor-associated macrophages, PD-L1, Lung cancer

## Abstract

Current immunotherapies represent a highly promising approach for the treatment of lung cancer. However, their clinical efficacy is often constrained by T cell exhaustion, antibody resistance, and poor tumor penetration, highlighting an urgent need for innovative therapeutic strategies. In this study, we developed an acidic-responsive liposome delivery nanocarrier (PEOz-liposome) for the co-delivery of a programmed cell death ligand 1 (PD-L1)-targeting antagonistic peptide and a Toll-like receptor 7/8 (TLR7/8) agonist. The resulting formulation, designated DRPL (DPPA+R848@PEOz-liposome), demonstrated TME weakly acid-dependent drug release, with cumulative release at pH 6.4 approximately threefold higher than that at physiological pH 7.4. DRPL effectively activated cytotoxic T lymphocytes, reprogrammed tumor-associated macrophages toward M1 phenotype, and promoted dendritic cells maturation. DRPL significantly enhanced tumor accumulation, markedly suppressed primary tumor growth, and remodeled the tumor microenvironment from an immunosuppressive to an immunoactivated state in lung tumor-bearing mice. Critically, DRPL elicited durable, antigen-specific immune memory, as evidenced by complete resistance to tumor rechallenge in cured mice. In summary, the acid-responsive liposomal platform for PD-L1 antagonistic peptide and TLR7/8 agonist codelivery, exhibited significant potential for the immunotherapy of lung cancer via ternary effects, offering a promising ternary therapeutic strategy for lung cancer.

## Graphical abstract

The acid-responsive liposomal immunoactivation system (DRPL) was engineered to co-deliver the TLR7/8 agonist R848 and a PD-L1 antagonistic peptide. This integrated nanoplatform combines dual immunomodulatory functions and demonstrates significant promise for lung cancer immunotherapy.

**Figure:**
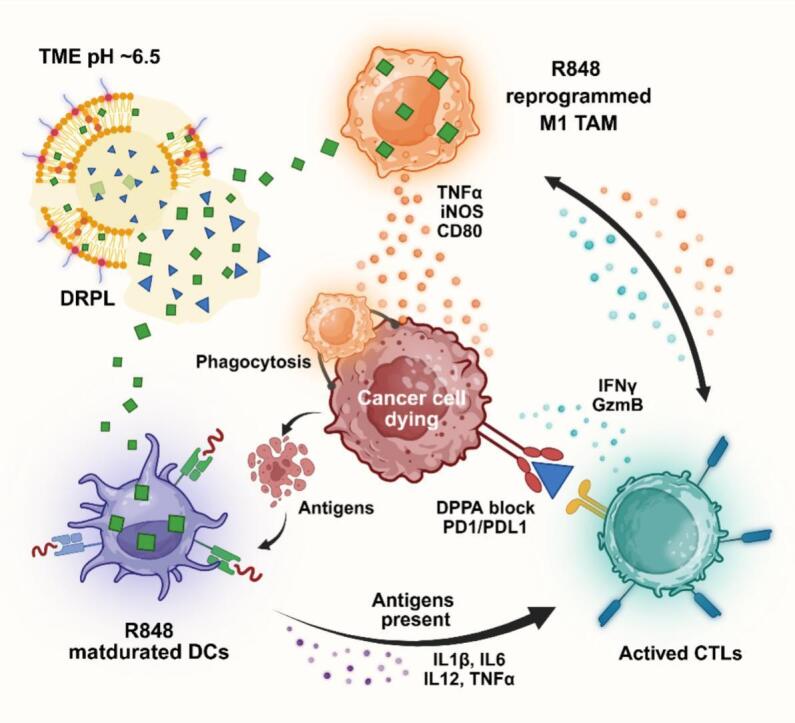

## 1 Introduction

Lung cancer is still one of the most prevalent and fatal malignancies worldwide, with a low five-year survival rate despite advances in chemotherapy, radiotherapy and molecular targeted therapy (Zhang et al., 2026; Liang et al., 2022; Siegel et al., 2024). In recent years, immunotherapy has been proven effective against lung cancer, prolonging survival time, and offering improved outcomes over conventional treatments by harnessing the immune system to recognize and eliminate cancer cells (Hendriks et al., 2024). Among immunotherapy approaches, immune checkpoint blockade targeting to the programmed death-1/programmed death-ligand 1 (PD-1/PD-L1) axis has achieved considerable clinical success (Zhao et al., 2025a). Antibody-based PD-1/PD-L1 inhibitors including atezolizumab, avelumab, and durvalumab, reinvigorate exhausted cytotoxic T lymphocyte cells (CTLs) and restore cytotoxic function (Qin et al., 2025). However, only 10–30% patients benefited from anti-PD-1/PD-L1 therapy despite the high-cost (Lin et al., 2024). Thus, alternative PD-L1 antagonists peptides need to be investigated. Compared with monoclonal antibodies, PD-L1 antagonist peptides offer several advantages as potential therapeutic agents, such as lower production costs, enhanced stability, reduced immunogenicity, and improved tumors penetration (Zhao et al., 2025b). DPPA, a selective PD-L1 blocking peptide (sequence NYSKPTDRQYHF), has shown great potential in immunotherapy by inhibiting tumor growth and extending survival in mice through immune activation (Chang et al., 2015; Yang et al., 2022).

The tumor immunity cycle proposes that CTLs cannot independently exert anti-tumor functions but rather complete this process through a series of coordinated networks with tumor-infiltrating lymphocytes in tumor microenvironment (TME) (Wang et al., 2024a). Tumor-associated macrophages (TAMs) and dendritic cells (DCs) play pivotal roles in modeling anti-tumor immunity (Polania et al., 2025). TAMs predominantly adopt a pro-tumoral M2-like phenotype, secreting cytokines such as Arg1, IL-10, TGF-β and VEGF, facilitating tumor proliferation, metastasis, invasion, and angiogenesis (Xu et al., 2025). Moreover, TAMs also inhibit CTLs function and DCs mature, recruit regulatory T cells (Tregs) and myeloid-derived suppressor cells (MDSCs), resulting in an immunosuppressive TME (Lin et al., 2023; Zhang et al., 2022). DCs presented tumor antigens to naive CTLs, thereby stimulating CTLs activation and proliferation, maintaining an immune response cycle during tumor evolution (Gehrcken et al., 2025; Ni et al., 2023). However, immunosuppressive TME restrains the migration and maturation of DCs, which accounts for the poor potency of PD-1/PD-L1 blockade.

Toll-like receptor 7/8 (TLR7/8) agonists have shown great potential in cancer treatment by reprogramming TAMs and maturating DCs (Zhang et al., 2025; Dai et al., 2025). Importantly, nanoparticles encapsulated TLR7/8 agonists have been reported to enhance the efficiency of anti-PD-1 or anti-PD-L1 antibody immunotherapy (Zhang et al., 2023a; Wang et al., 2025a). Nevertheless, few studies encapsulate TLR7/8 agonists and PD-L1 antagonist peptides in the same nanocarrier-based delivery system to address off-target effect, inefficient collaboration and multiple administrations. Liposomes are versatile nanocarriers that are capable of co-delivering hydrophilic and hydrophobic agents with minimal toxicity (Huang et al., 2023; Moloudi et al., 2024a; Moloudi et al., 2024b). Notably, responsive liposomes had designed to release payloads under specified conditions in TME, such as acidic, high level of reactive oxygen, and MMP2 enzyme (Ashrafizadeh et al., 2022; Wang et al., 2024b).

**Scheme 1:**
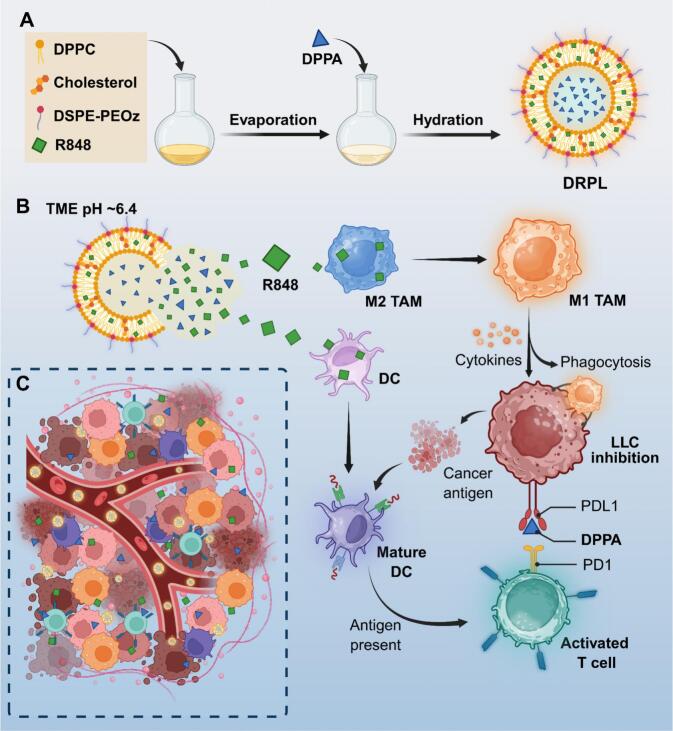
Schematic illustration of the preparation and biological effect of DRPL. (A) The preparation of DRPL. (B) DRPL reprogrammed TAM, maturated DC and activated CD8+ T cells by responsively releasing R848 and DPPA in TME. (C) The remodeling of TME after DRPL treatment.

In this study, we developed an acidic-responsive liposomal therapeutic agent (DPPA + R848@PEOz-liposome, DRPL) for the co-delivery of PD-L1 antagonistic peptide DPPA and TLR7/8 agonist Resiquimod (R848) to the tumor site. DRPL composed of DPPC, cholesterol, and DSPE-PEOz2000, formed a stable liposomal structure with DPPA encapsulated in the inner hydrophilic phase and R848 embedded in the lipid bilayer (Scheme 1A). Upon accumulation in tumors via the enhanced permeability and retention effect, DRPL disintegrated in the acidic TME. DPPA released from DRPL bound to PD-L1 on Lewis lung cancer cells (LLCs), thereby inhibiting PD-1/PD-L1 immune checkpoint signaling and restoring. Concurrently, R848 triggered repolarization of TAMs toward an antitumor M1-like phenotype and promoted DCs maturation, resulting in enhanced cancer cells death and improved antigen presentation (Scheme 1B). By coordinately activating CTLs, reprogramming immunosuppressive TAMs, and maturing DCs, this combinatorial liposomal platform provides a rationally designed and therapeutically effective approach to remodel the immunosuppressive TME and potentiate antitumor immunity in lung cancer (Scheme 1C).

## 2 Materials and methods

### 2.1 Materials

Dipalmitoyl phosphatidylcholine (DPPC, R-DPPC-003), Cholesterol (R-cholesterol), and 1,2-Distearoyl-sn-glycero-3-phosphorylethanolamine-Poly (2-Ethyl-2-Oxazoline) (DSPE-PEOz2000, R-PL2056) were obtained from Ruixi Biological Technology (Xi'an, China). The PD-L1 blocking peptide NYSKPTDRQYHF (DPPA) was obtained from Jier Biotechnology (202,562, Shanghai, China). The Toll-like receptor 7/8 agonist Resiquimod (R848) was purchased from Selleck Chemicals (S8133, Shanghai, China). The bicinchoninic acid (BCA) protein assay kit was obtained from Pierce Biotechnology (A55864, California, USA). Dulbecco's Modified Eagle Medium (DMEM, C11995500BT), Roswell Park Memorial Institute (RPMI) 1640 medium (11875119), fetal bovine serum (FBS, 10099-141C), macrophage colony-stimulating factor (M-CSF, PMC2044), and penicillin-streptomycin (15140–122) were all purchased from Gibco (New York, USA). Antibodies against F4/80 (111603), CD80 (104725), and CD86(159219) were purchased from BioLegend (California, USA). Calcein-AM/PI staining kit (C2015S), Cell Counting Kit-8 (CCK-8, C0037), and Hoechst 33342 (C1022) were purchased from Beyotime Biotechnology (Jiangsu, China).

### 2.2 Preparation of liposomes

Liposomes were prepared using the thin-film hydration method (Moloudi et al., 2024a; Moloudi et al., 2024b). Briefly, DPPC, cholesterol, and DSPE-PEOz at a molar ratio of 2:1:0.33 was mixed with R848 (2.5%, 5%, 10%, and 15% of the weight of liposome components), and then dissolved in trichloromethane and methanol (3:1, *v*/v). The organic solvent was removed by rotary evaporation to form a uniform lipid film. The film was then hydrated with a pH 7.4 buffer containing DPPA in different dosages for 30 min. To reduce particle size and achieve uniformity, the liposomal suspension was sequentially extruded 20 times through polycarbonate membranes with pore sizes of 400 nm, 200 nm, and 100 nm using a mini-extruder (Avestin, Canada). Unencapsulated R848 and DPPA were removed by centrifugation at 10,000 *g* for 30 min. The final product, acidic-responsive liposomes co-loaded with DPPA and R848 (DPPA+R848@PEOz-liposomes, DRPL) was collected for further use.

### 2.3 Characteristics of DRPL

The transmission electron microscopy (JEOL JEM1400PLUS, Japan) was used to test the morphological characteristics of DRPL. Particle size and zeta potential were determined by Zetasizer Nano ZS90 (Malvern, UK).

To optimize the encapsulation efficiency (EE), DPPA@PEOz-liposomes (DPL) and R848@PEOz-liposomes (RPL) were prepared at varying drug-to-lipid mass ratios (2.5%, 5%, 10%, and 15%) using thin-film hydration as described above. Un-encapsulated DPPA and R848 were separated from liposomes by centrifugation. The concentrations of unencapsulated drugs in the supernatant were quantified using a UV-2600 ultraviolet spectrophotometer (Shimadzu, Japan). The drug loading efficiency (LE) and encapsulation efficiency (EE) were calculated using the following equations:

$$\mathrm{LE}\;\left(\%\right)=\left(\mathrm{Wa}-\mathrm{Wu}\right)/\mathrm{Wt}\times 100\%$$

$$\mathrm{EE}\;\left(\%\right)=\left(\mathrm{Wa}-\mathrm{Wu}\right)/\mathrm{Wa}\times 100\%$$

Where Wᵤ represents the amount of unencapsulated drug in the supernatant, and W_a_ is the total amount of drug initially added, and Wₜ is the total weight of liposomes.

### 2.4 Cell culture and induction

The mouse Lewis lung carcinoma (LLC) cell line was purchased from the Shanghai Institute of Cell Biology (Shanghai, China). LLC cell line was maintained in DMEM containing 10% FBS and 100 U/mL penicillin-streptomycin, under standard culture conditions (37 °C, 5% CO₂). To induce PD-L1 upregulation, the cells were exposed to 10 ng/mL interferon-gamma for 24 h.

The femurs and tibias from 6-week-old C57BL/6 mice were stripped of muscle tissue, and cut into pieces followed rinse for 10 times. After filter by 40-μm cell strainer, the suspension was centrifuged at 2000 rpm for 5 min. The single cell sediment was resuspended in red blood cell lysis buffer at room temperature for 5 min to remove erythrocytes. Subsequently, cells were resuspended in RPMI-1640 medium with 10% FBS, 20 ng/mL M-CSF, and 100 U/mL penicillin-streptomycin and supplemented fresh medium on Day 3. On day 5, BMDMs were polarized toward an M2-like tumor-associated macrophage phenotype using 20 ng/mL IL-4 for 48 h. Adherent cells were identified by Arg1 and CD206 through qRT-PCR. Similarly, bone marrow-derived dendritic cells (BMDCs) were isolated as above method and induced by 20 ng/mL GM-CSF and 10 ng/mL IL-4 for 7 days. Fresh medium containing M-CSF was replenished on Day 4. BMDCs were collected from culture medium by centrifugation at 2000 rpm for 5 min and characterized by CD11c.

### 2.5 In vitro PD-L1 blockade in LLC cells

LLC cells were stimulated with 10 ng/mL IFNγ for 24 h to increase PD-L1 expression in vitro. LLC cells were treated with PBS, DPPA+R848 (designated as DR), DPL, RPL, and DRPL in a pH 6.4 culture medium, or DRPL in pH 7.4 culture medium (designated as DRPL 7.4) for 12 h. Subsequently, cells were incubated with APC-conjugated anti-PD-L1 antibody (124311, Biolegend) and analyzed by flow cytometry. In a parallel experiment, the treated LLC cells were co-cultured with CTLs preactivated by phorbol myristate acetate (100 ng/mL) and ionomycin (1000 ng/mL) for 4 h. The CTLs were then collected and stained with PE-conjugated anti-CD69 antibody (164203, Biolegend) to evaluate their activation status. LLC cells were stained with Annexin V-FITC/PI for apoptosis analysis.

### 2.6 Cell apoptosis

LLC cells, BMDMs and BMDCs were seed in 12-well plates and treated with PBS, DR, DPL, RPL, DRPL, or DRPL 7.4 for 24 h. Cells were washed twice with PBS (10010023, Gibco,) and digested with trypsin without Ethylenediaminetetraacetic acid (EDTA, 15050065, Gibco,). Cells were centrifuge at 2000 rpm for 5 min and stained by Annexin V-FITC/PI (C1062S, Beyotime) for 15 min at room temperature. Cells were kept on ice waiting to be analyzed by a flow cytometer (CytoFLEX S.4, Beckman). The statistics were processed by FlowJo V10.8.1.

### 2.7 Quantitative real-time PCR (qRT-PCR) analysis

IL-4-primed BMDMs or BMDCs were treated for 24 h with PBS, DR, DPL, RPL, DRPL, or DRPL 7.4. After incubation, total RNA was isolated with TRIzol reagent (Invitrogen, USA). Subsequent RT-qPCR was performed to analyze the expression levels of different genes. Primer sequences are showed in Table S1.

### 2.8 The biodistribution in vivo

Male C57BL/6 mice (6 weeks old, ∼25 g) were obtained from Vital River Laboratory Animal Technology Co., Ltd. (Beijing, China). All the animal experiments were performed in accordance with the guidelines approved by the Institutional Animal Care and Use Committee of Guangzhou Medical University (GY2024–033). Tumor-bearing mice were generated by subcutaneous injection of 3 × 10^6^ LLC cells suspended in 200 μL PBS into the right shoulder of male C57BL/6 mice. When tumor volumes reached approximately 100 mm^3^, the mice were randomly assigned to three groups and received intravenous administrations of saline, DiR, or DRPL. The latter was fluorescently labeled with the near-infrared dye DiR. The in vivo distribution of each formulation was tracked at designated time points using an in vivo imaging system (PerkinElmer, USA). Throughout imaging, anesthesia was maintained with 2% isoflurane. Following the final imaging session, tumors and major organs were excised for ex vivo fluorescence imaging and subsequent quantitative analysis using the system's integrated software.

### 2.9 Circulation lifetime

Healthy C57BL/6 mice were administrated with saline, free DiR, or DiR-labeled DRPL through intravenous injection. Blood samples (10 μL) were collected by tail clipping at predetermined time points (1, 2, 4, 6, 8, 12, 24, and 48 h), diluted with 10 mM EDTA solution, and analyzed using a fluorescence imaging system (PerkinElmer, USA) to determine the circulation profile of DiR-labeled formulations.

### 2.10 Tumor inhibition in vivo

Tumor-bearing mice received intravenous injections of saline, free DPPA combined with R848 (DR), DPL, RPL, or DRPL every three days (R848 equivalent to1.5 mg/kg). Tumor dimensions and body weight were recorded every two days. Tumor volume (V) was determined using the formula V = (a^2^ × b)/2, where a and b denote the tumor width and length, respectively. Tumor-bearing mice at Day 19 were surgically to remove the primary tumors, and subcutaneously injected LLC cells in the other axilla. After 7 days, the development of rechallenged tumors was monitored each 2 days for 5 times. After the animal experiments were finished, all mice were euthanized, and samples of tumors, blood, and major organs were collected. The excised tumors were photographed, fixed in 4% paraformaldehyde, paraffin-embedded, and sectioned for staining with TUNEL, Ki67, CD3e, and iNOS/Arg-1. Blood samples were assessed for biochemical markers such as alanine transaminase (ALT), aspartate transaminase (AST), creatinine (CREA), and blood urea nitrogen (BUN). Major organs (heart, liver, spleen, lung, and kidney) were processed for hematoxylin and eosin (H&E) staining to evaluate histopathological changes.

To assess immune cell infiltration, tumors were digested with collagenase IV to obtain single-cell suspensions; after filtration and red blood cell lysis, cells were stained with antibody panels targeting CD45/F4/80/CD206/CD80 for macrophages, CD45/CD3e/CD8/CD69 for activated CD8^+^ T cells, CD45/CD3e/CD4/FOXP3 for regulatory T cells (Tregs), and CD45/CD11c/MHC II for mature dendritic cells.

### 2.11 Statistical analysis

All data are expressed as mean ± standard deviation. Statistical analysis was performed using Student's *t*-test or one-way ANOVA in GraphPad Prism 8.0 (GraphPad Software, USA). * *P* < 0.05, ** *P* < 0.01, and *** *P* < 0.001 were considered statistically significant.

## 3 Results

### 3.1 Preparation and characterization of DRPL

**Fig. 1:**
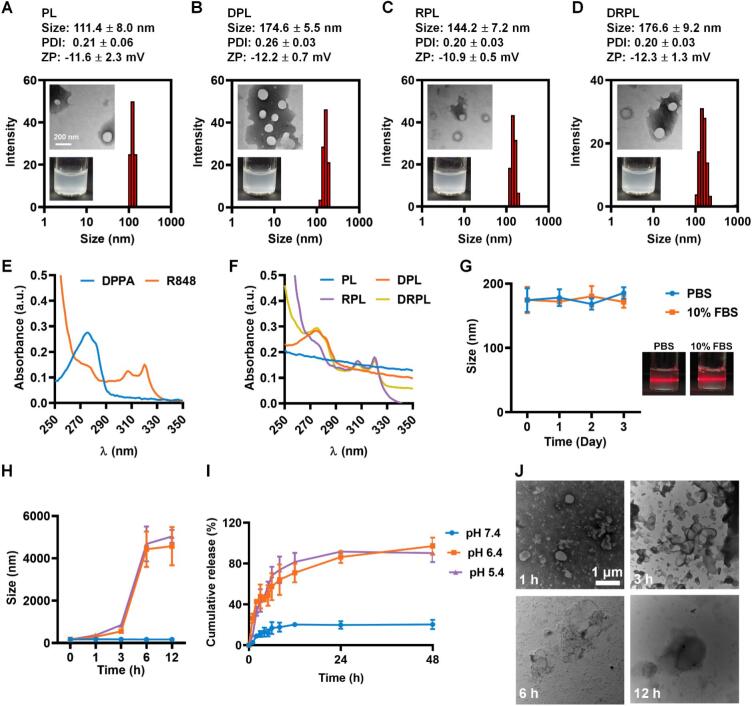
Optimization and characterization of DRPL. (A) Particle size distribution and TEM images of liposomes, (B) DPL, (C) RPL, and (D) DRPL. (E) UV–Vis analysis of R848 and DPPA, Scale bar, 200 nm. (F) UV–Vis analysis of liposome-based nanoparticles. (G) Particle size changes of DRPL in PBS and 10% FBS over 72 h. (H) Cumulative drug release profiles of DRPL at pH 5.4, 6.4, and 7.4 buffers. I Particle size of DRPL incubated in different pH buffers within 12 h. (J) TEM images illustrating morphological changes of DRPL incubated in pH 6.4 buffer within 12 h. Scale bar, 1 μm. Data are represented as mean ± SD, *n* = 3.

The thin-film hydration method was used to prepare the cargo-loaded liposomes. First, the agonist R848 and lipid components, including DPPC, cholesterol, and DSPE-PEOz2000, were dissolved in a mixture of trichloromethane and methanol (3:1, *v*/v). The solvent was then removed by rotary evaporation, forming a thin film on the round-bottom flask. Next, the PD-L1antagonistic peptide DPPA was dissolved in water and added to the flask, where it was subjected to vibration and ultrasonication to form liposomes. The weight ratio of DPPA to liposome was screened. A weight ratio of 10% yielded the encapsulation efficiency of over 50% and loading efficiency of ∼5% (Fig. S1A). Similarly, the encapsulation efficiency of R848 exceeded 90% and loading efficiency of ∼10% (Fig. S1B). Considering these results, we selected a 10% weight ratio of either R848 or DPPA to liposome for preparing the cargo-loaded formulation. Non-cargo-loaded liposomes (named PEOz-liposomes) showed a size of 111.4 nm and a zeta potential of −11.6 mV (Fig. 1A), whereas DPPA-loaded liposomes (DPL) and R848-loaded liposomes (RPL) exhibited an increased size of 174.6 nm and 144.2 nm, respectively (Fig. 1B and C). DPPA and R848-loaded liposomes exhibited a spherical morphology with a diameter of 176.6 nm and a zeta potential of −12.3 mV, along with a particle distribution index (PDI) of 0.20 (Fig. 1D). The UV–Vis spectrum analysis indicated that DPPA and R848 showed the characteristic absorption peaks at ∼270 nm and ∼ 325 nm, respectively (Fig. 1E). Moreover, the characteristic absorption peaks of DPPA and R848 existed still after the encapsulation with liposomes, and DRPL possessed the ones of both DPPA and R848 (Fig. 1F), confirming the successful loading of both components. DRPL dispersed in PBS and 10% FBS showed no obvious size changes over 3 days, confirmed by the Tyndall effect (Fig. 1G), implying a good stability.

To evaluate the acidic responsiveness, DRPL were incubated in different pH environments (pH 7.4, 6.4, and 5.4). At pH 6.4, the size of nanoparticles increased markedly to 5000 nm within 12 h, while at pH 7.4, only slight size changes were observed (Fig. 1H). The cumulative drug release profile showed that DRPL released drugs effectively at pH 6.4, reaching ∼75% release at 6 h and nearly 100% at 12 h, which was much higher compared with the one at pH 7.4 (Fig. 1I). DRPL exhibited a progressive increase in particle size over time under acidic conditions (pH 6.4), characteristic of gradual disassembly (Fig. 1J), which supports its potential for acidic-responsive drug release in the tumor microenvironment. This acidic responsiveness confers controlled release properties appropriate for tumor environments, which typically have a mildly acidic pH of around 6.4. Nanoparticles with reduced protein absorption are preferred to avoid protein-mediated biodistribution effects (Kong et al., 2024). DRPL exhibited a bovine serum albumin (BSA) adsorption at ∼10 μg/mg, which was ∼3-fold lower than that of cationic liposomes, indicating favorable stability under physiological conditions (Fig. S2). Furthermore, DRPL exhibited minimal hemolytic activity within the working concentration range (Fig. S3A and S3B). Overall, these results demonstrate that DRPL exhibited significant acidic responsiveness and enhanced drug release under acidic conditions, making it well-suited for targeted drug delivery in tumor.

### 3.2 In vitro biological effect of DRPL

**Fig. 2:**
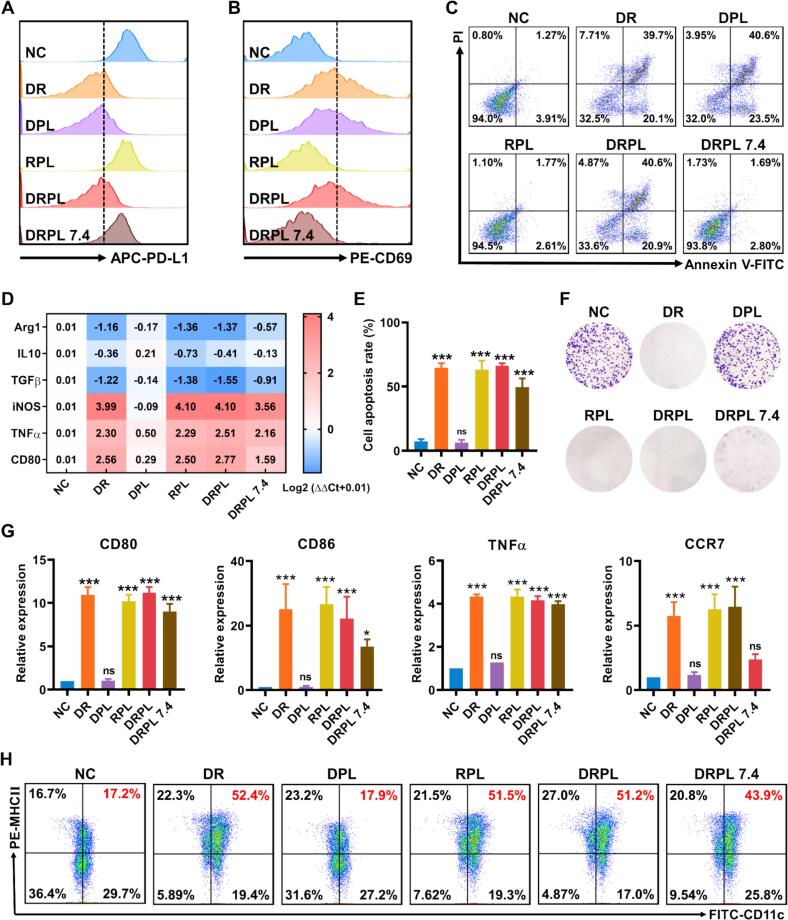
In vitro biocompatibility and immune responses of DRPL. (A) PD-L1 blockade of LLC after different treatments. (B) T cells activation after coculturing with LLC pretreated with different formulations. (C) Apoptosis of LLC pretreated with different formulations after coculturing with T cells for 24 h. (D) Relative mRNA levels of BMDMs treated with PBS, DR, DPL, RPL, DRPL, or DRPL 7.4. (E) Cell apoptosis of LLC after coculturing with different treated BMDMs. (F) Colony formation of LLC cocultured with different treated BMDMs. (G) Relative mRNA expressions of BMDCs after PBS, DR, DPL, RPL, DRPL, or DRPL 7.4 treatments. (H) Maturation of BMDCs with different treatments. NC, PBS; DR, DPPA + R848; DPL, DPPA @PEOz-liposome; RPL, R848@PEOz-liposome; DRPL, DPPA+R848@PEOz- liposome. Data are represented as mean ± SD, *n* = 3; *P < 0.05, ** *P* < 0.01, and ***P < 0.001 vs. NC.

IL4 induced BMDMs highly expressed Arg1 and IL10 in mRNA levels (Fig. S4A). 86.0% BMDCs were CD45 and CD11c positive (Fig. S4B). Then we evaluated the biological effect induced by DRPL in cells. Annexin V-FITC/PI staining assays revealed that DRPL did not induce significant apoptosis in LLC cells, BMDM, and BMDC (Fig. S5A–C), supporting its potential safety for application. Upon treatment with different formulations (PBS, DR, DPL, RPL, DRPL, or DRPL 7.4), DPL and DRPL exhibited comparable PD-L1 blockade to free DPPA+R848 in pH 6.4 (Fig. 2A), implying the effective acidic-responsive disintegration of PEOz-liposome. Whereas DRPL in normal medium (DRPL 7.4) showed negligible PD1/PD-L1 blockade efficacy because of the encapsulated DPPA (Fig. 2A), illustrating the essential of disintegration of PEOz-liposome in acidic tumor microenvironment. Additionally, DRPL showed similar efficiency in PD-L1 blockade as anti-PD-L1 antibody (Fig. S6A). Subsequently, LLC treated with DRPL were cocultured with preactivated CTLs for 24 h. Profiting by the effective release of DPPA from DRPL under acidic conditions, the PD-1/PD-L1 axis was effectively blocked, thereby leading to activation of CTLs substantially, evidenced by the increase of CD69-positive cells (Fig. 2B). Activated CTLs induced approximately 60% LLC apoptosis (Fig. 2C). Likewise, DRPL exhibited parallel capability on CTLs activation and anti-tumor activity to anti-PD-L1 antibody in vitro (Fig. S6B and S6C). The results demonstrated that DRPL showed effective PD-1/PD-L1 blockade and CTLs activation in acidic environments, exerting preferable inhibition of lung cancer cells.

Simultaneously, BMDMs treated with R848-containing formulations showed significant upregulation of iNOS, CD80, and TNF-α mRNA levels, accompanied by downregulation of Arg1, IL-10, and TGF-β (Fig. 2D and Fig. S7), suggesting efficient repolarization of M2 macrophages toward a pro-inflammatory M1 phenotype. Both RPL and DRPL demonstrated similar repolarization efficiency with DR, implying the acidic environment triggered the release of R848 from PEOz-liposomes. DRPL 7.4-treated BMDMs also showed efficacy in macrophage repolarization (Fig. 2D and Fig. S6), which may because of a certain cellular uptake of DRPL by BMDMs during 24 h incubation. Then DRPL-treated BMDMs induced 66.2% LLC apoptosis after coculture for 48 h (Fig. 2E and Fig. S8), suggesting effective tumor inhibition of DRPL in TME. Besides, BMDMs repolarized by DRPL showed a 2-fold increase in phagocytic efficiency against GFP-LLC cells after coculture (Fig. S9). Analogously, LLC colony formation was significantly inhibited by DRPL repolarized BMDMs (Fig. 2F and Fig. S10). Collectively, M2 macrophages were repolarized to a pro-inflammatory phenotype by DRPL in a simulated acidic TME condition, contributing to the suppression of lung cancer cells via apoptosis induction and phagocytosis. BMDCs treated with R848-containing formulations showed enhanced maturation with low cytotoxicity, as evidenced by increased expression of CD80, CD86, TNFα and CCR7 (Fig. 2G). DRPL further increased the expressions of IL1β, IL6, and IL12 while reducing IL10 expression (Fig. S11). Moreover, 51.2% of BMDCs treated with DRPL upregulated MHC-II expression (Fig. 2H), the key molecule in antigen presentation. Consistently, these results demonstrated that DRPL effectively activated CTLs, repolarized TAMs, and promoted DCs maturation, highlighting its potential as a multiple-mode immunotherapy for lung cancer.

### 3.3 In vivo imaging and circulation lifetime

**Fig. 3:**
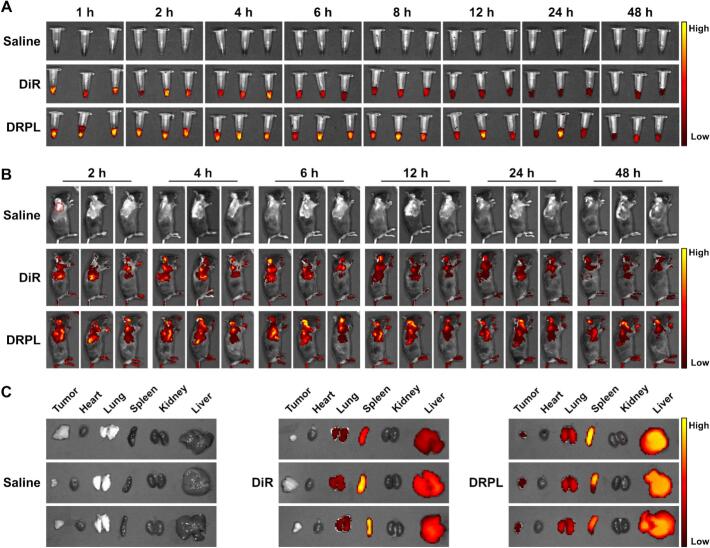
Circulation kinetics and biodistribution of DRPL in vivo. (A) Fluorescence imaging of blood samples collected at indicated time points post-injection. (B) In vivo fluorescence imaging of tumor-bearing mice administrated with different formulations. (C) Ex vivo fluorescence imaging of excised tumors and major organs at 48 h post-injection.

We detected the circulation lifetime and biodistribution of the nanoparticles. Firstly, the circulation kinetics of DRPL were assessed in healthy mice. DiR and DRPL were administered intravenously into healthy C57BL/6 mice, and blood samples were collected at various time points. DRPL-treated mice maintained strong fluorescence signals for up to 48 h, demonstrating the prolonged circulation time, whereas the fluorescence intensity in the free DiR group decreased sharply within 6 h after injection (Fig. 3A), which was confirmed by quantitative analysis of blood fluorescence (Fig. S12). The data indicated that DRPL showed a good circulation properties, which might be beneficial for the in vivo cancer therapy.

To evaluate the in vivo biodistribution of DRPL, the liposomes were labeled with the near-infrared dye DiR. The LLC cell tumor-bearing mouse model was generated by subcutaneous injection of LLC cells into the right shoulder. After intravenous injection of DiR-labeled DRPL, a pronounced fluorescence signal was detected at tumor sites as early at 2 h post-administration and persisted for up to 48 h, demonstrating significantly higher intensity compared to the free DiR group (Fig. 3B). Ex vivo imaging of resected tumors and major organs further revealed substantially enhanced tumor accumulation in the DRPL-treated group (Fig. 3C). Quantitative assessment of fluorescence intensity across organs supported the tumor-targeting propensity of DRPL (Fig. S13). These results indicate that DRPL effectively accumulates in tumor tissue, suggesting its potential for effective tumor inhibition. Collectively, these findings demonstrate that DRPL exhibits favorable pharmacokinetics and tumor-targeting capability, likely attributed to its liposomal architecture, which facilitates prolonged circulation and enhanced tumor accumulation, supporting its potential for effective lung cancer therapy.

### 3.4 Primary tumor inhibition

**Fig. 4:**
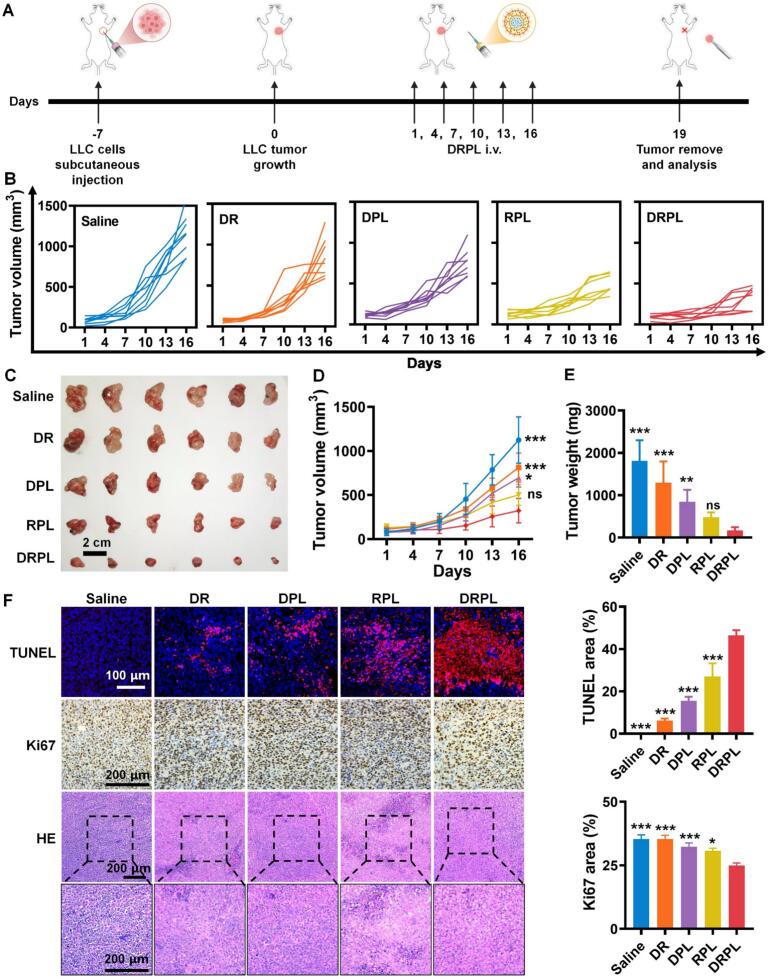
Therapeutic efficacy of different formulations in LLC tumor-bearing mice. (A) Schematic timeline of the in vivo treatment schedule. (B) Tumor volume progression in each group during treatment. (C) Representative images of tumors excised from mice after treatment. Scale bar, 1 cm. (D) Final tumor volumes in each group. (E) Excised tumor weights. (F) TUNEL staining, Ki67 and H&E of tumors from mice. Scale bar, 100 μm. DR, DPPA + R848; DPL, DPPA @PEOz-liposome; RPL, R848@PEOz-liposome; DRPL, DPPA+R848@PEOz-liposome. The data was represented as mean ± SD, *n* = 6; *P < 0.05, **P < 0.01, and ***P < 0.001 vs. DRPL.

The antitumor efficacy of different formulations was evaluated in LLC tumor-bearing mice. Mice received intravenous injections of saline, DR, DPL, RPL, or DRPL every 3 days for a total of 6 times (Fig. 4A). Among all groups, DRPL exhibited the strongest tumor growth inhibition (Fig. 4B and C). The average tumor volume in the DRPL-treated group was approximately one-sixth that of the saline group (Fig. 4D). Although DPL and RPL showed moderate tumor-suppressive effects, their efficacy was 2–3-fold lower than that of DRPL (Fig. 4C and D). This trend was further confirmed by significantly reduced tumor weight in DRPL-treated mice (Fig. 4E). TUNEL staining revealed the most pronounced cancer cell apoptosis in DRPL group, as evidenced by strong red fluorescence signals, with 46.5 ± 2.0% TUNEL-positive cells (Fig. 4F). Consistently, immunostaining showed markedly decreased Ki67 expression in tumors from DRPL-treated mice, indicating substantial inhibition of tumor cell proliferation (Fig. 4F). H&E staining further revealed nuclear pyknosis in tumors from DRPL-treated mice (Fig. 4F). Throughout the treatment period, no significant changes in body weight were observed across groups, suggesting good systemic tolerability (Fig. S14). Collectively, these findings demonstrated that DRPL exerted potent antitumor activity with minimal toxicity, underscoring its promise as a therapeutic strategy for lung cancer.

### 3.5 The remodeling of the tumor microenvironment

**Fig. 5:**
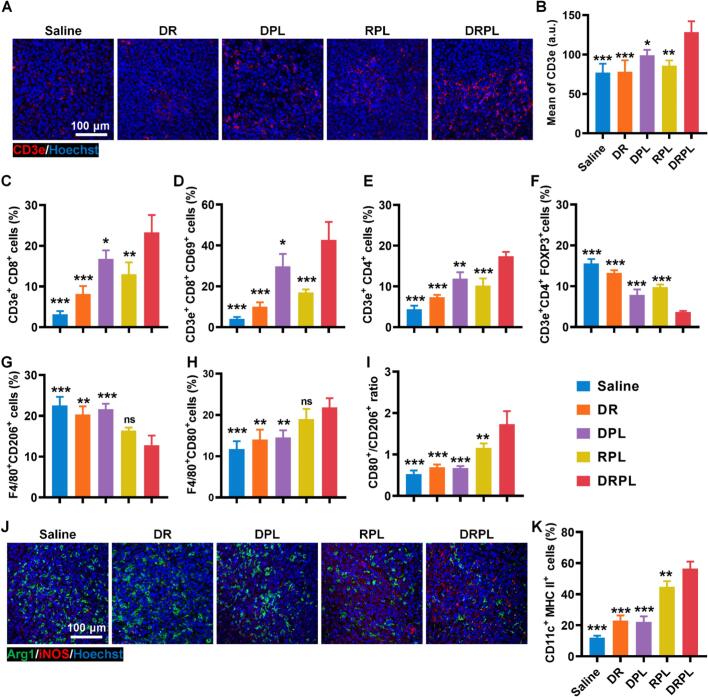
Immunomodulation of DRPL in TME. (A) Immunofluorescence of T cells in tumor. (B) Quantitative analysis of CD3e positive areas. (C) Quantification of CD8^+^ T, (D) activated CD8^+^ T cells, (E) CD4^+^ T cells, (F) Treg, (G) M2 TAMs and (H) M1 TAMs in tumors. (I) Ratios of M1/M2 TAMs. (J) Immunofluorescence staining of Arg1 and iNOS in tumor; K Quantification of mature DCs in tumors. DR, DPPA+R848; DPL, DPPA@PEOz-liposome; RPL, R848@PEOz-liposome; DRPL, DPPA+R848@PEOz-liposome. Data are represented as mean ± SD; * *P* < 0.05, ** *P* < 0.01, and *** *P* < 0.001 vs. DRPL.

The immunomodulatory effects of DRPL on the tumor microenvironment were further evaluated. Instead, the infiltration of T cells increased significantly (Fig. 5A and B). FACS analysis revealed that the DPPA-containing liposomal platforms (DPL and DRPL) significantly increased the proportion of CD8^+^ cells, reaching 7.62-fold higher than the saline group and 2.16-fold higher than the DR group (free DPPA+R848, Fig. 5C and Fig. S15A), demonstrating the efficient blockage of PD-L1 by DPPA. Correspondingly, 43.3% of CD8^+^ T cells were activated in DRPL-treated mice, consistent with PD-1/PD-L1 axis blockade (Fig. 5D and Fig. S15B). The proportion of CD4^+^ T cells also increased from 4.79% to 18.4% (Fig. 5E and Fig. S15C), whereas Tregs (CD3e^+^CD4^+^FOXP3^+^) were reduced by 3.92-fold (Fig. 5F and Fig. S15D).

The treatment with DRPL also promoted repolarization of tumor-associated macrophages (TAMs), with CD206^+^ cells decreasing from 23.4% in the saline group to 15.4% (Fig. 5G and Fig. S16A). In parallel, the proportion of CD80^+^ macrophages a marker of the pro-inflammatory M1 phenotype, increased from 11.1% in the saline group to 20.5% following DRPL treatment (Fig. 5H and Fig. S16B), suggesting that the successful delivery of R848 contributed to TAM repolarization. The ratio of CD80^+^/CD206^+^ macrophages in DRPL-treated group increased 3.3-fold compared with NC (Fig. 5I). Immunofluorescence staining further confirmed the M2-to-M1 repolarization of TAMs, as evidenced by reduced green (M2) and enhanced red (M1) fluorescence in the DRPL group (Fig. 5J). FACS analysis further demonstrated a marked increase in MHC-II^+^ dendritic cells in the DRPL group (55.0%) compared with the saline group (12.1%) (Fig. 5K and Fig. S16C), implying the enhanced antigen presentation capacity. Collectively, these results demonstrated that DRPL effectively remodeled the immunosuppressive tumor microenvironment into a pro-inflammatory one, thereby contributing to its potent therapeutic efficacy.

### 3.6 Rechallenged tumor inhibition

**Fig. 6:**
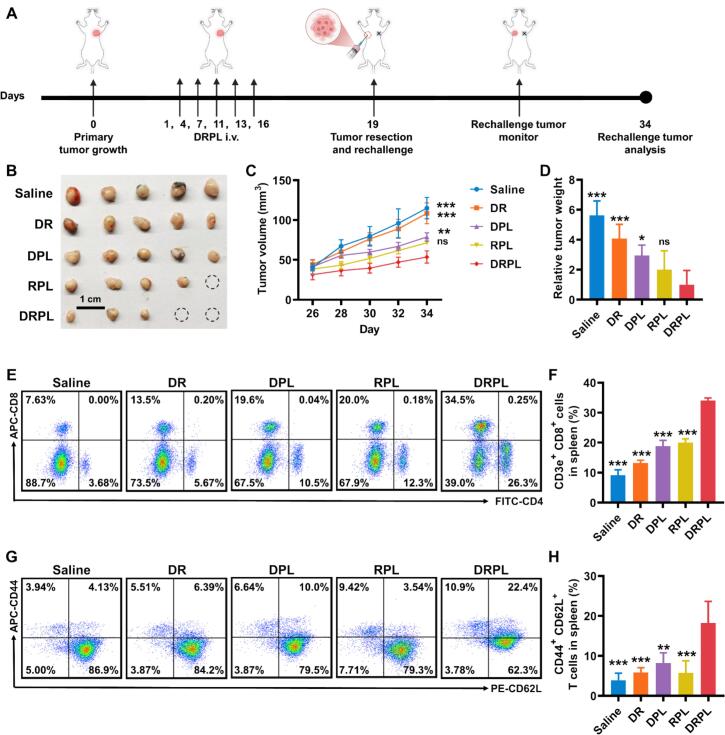
Rechallenged tumors inhibition of DRPL. (A) Schematic timeline of rechallenge tumors analysis. (B) Representative images of rechallenge tumors after treatment. (C) Volume of rechallenge tumors; (D) Weight of rechallenge tumors. (E) Quantification of CD8^+^ and CD4^+^ T cells in spleens. (F) Statistic comparison of the amount of CD8^+^ T cells. (G) Status of T cells in spleens analyzed by FACS. (H) Statistic comparison of CD44^+^CD62L^+^ T cells. DR, DPPA+R848; DPL, DPPA@PEOz-liposome; RPL, R848@PEOz-liposome; DRPL, DPPA+R848@PEOz-liposome. Data are represented as mean ± SD; *P < 0.05, **P < 0.01, and ***P < 0.001 vs. DRPL.

As reported, 20–40% of stage I lung cancer patients experienced tumor recurrence after surgical resections, with a low 5-year post-recurrence survival of 15% (Wang et al., 2025b). Inducing and maintaining tumor-specific immune memory response is the core immunological effect for preventing recurrence, reducing metastasis, and achieving clinical cure after. Dendritic cells present tumor antigens to CTLs, thereby activating CTLs. Some of CTLs differentiate into effector T cells and kill tumor cells directly. While other CTLs differentiate into memory T cells, including central memory T cells (Tcm), effector memory T cells (Tem), and tissue-resident memory T cells (Trm). These memory T cells continuously surveil for tumor recurrence and metastasis. Upon re-encounter with cognate tumor antigen, memory T cells proliferate and differentiate into effector T cells rapidly, achieving a stronger immune response and effectively eliminate tumor cells. Immune memory is beneficial for tumor recurrence prevention. To evaluate the tumor immune memory capability induced by DRPL, rechallenged tumor mouse model was established to simulate recurrence after surgery. Tumor-bearing mice at Day 19 were surgically to remove the primary tumors and subcutaneously injected LLC cells in the other axilla. After 7 days, the development of rechallenged tumors was monitored each 2 days for 5 times (Fig. 6A). The excised rechallenged tumors in DRPL-treated mice exhibited the smallest volumes and slowest tumor growth compared with other formulations (Fig. 6B-D), demonstrating excellent sustained immune response. The spleens were collected for further analysis. Both CTLs (CD8^+^) and CD4^+^ T cells in spleen were significantly increased in DRPL group (Fig. 6E-F and S16), revealing the activation of host general immunity. Central memory T cells (Tcm) located in secondary lymphoid organs proliferate and differentiate into effector cells upon antigenic stimulation. The amount of CD44^+^CD62L^+^ T cells (Tcm) was increased from 4.13% to 22.4% (Fig. 6G and H). Correspondingly, the quantity of CD44^+^CD62L^−^ T cells (Tem) increased by 2.76-fold (Fig. 6G and S17A). While naïve T cells (CD44^−^CD62L^+^) decreased from 86.9% to 62.3% (Fig. 6G and S17B). These results illustrated that DRPL could induce a sustained immune response to inhibit tumor recurrence.

### 3.7 The biosafety in vivo

**Fig. 7:**
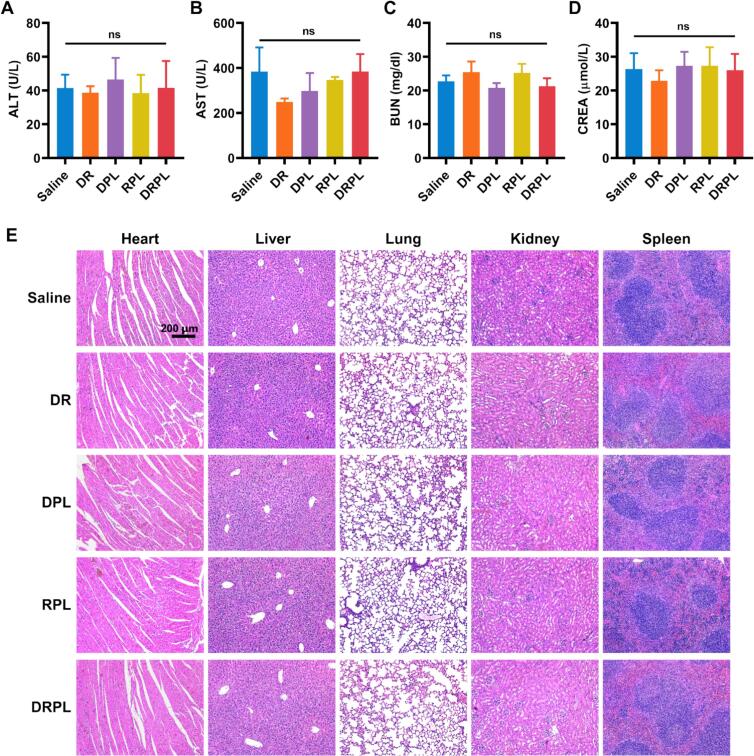
In vivo biosafety evaluation of different formulations. (A) ALT, (B) AST, (C) BUN, and (D) CREA level in mice after treatment with various formulations. (E) Representative H&E-stained images of major organs (heart, liver, spleen, lung, and kidney) collected at the end of the in vivo study. DR, DPPA + R848; DPL, DPPA @PEOz-liposome; RPL, R848@PEOz-liposome; DRPL, DPPA+R848@ PEOz-liposome. Data are represented as mean ± SD, *n* = 5; *P < 0.05, **P < 0.01, and ***P < 0.001 vs. Saline. ns, not significant. Scale bar, 200 μm.

To assess the in vivo biosafety of the formulations, key hematological and histopathological parameters were evaluated after treatment. Upon completion of the therapeutic regimen, the blood was obtained to measure liver and kidney function indicators, including alanine aminotransferase (ALT), aspartate aminotransferase (AST), blood urea nitrogen (BUN), and creatinine (CREA). No significant changes were detected across the saline, DR, DPL, RPL, and DRPL groups (Fig. 7A-D), suggesting minimal hepatorenal toxicity. Moreover, H&E-stained sections of heart, liver, spleen, lung, and kidney revealed no signs of inflammation, necrosis, or other pathological alterations (Fig. 7E). Overall, these findings indicate that the acid-responsive liposomal co-delivery system exhibits favorable biocompatibility and excellent in vivo safety profiles.

## 4 Discussion

Immunotherapy has revolutionized cancer treatment in recent years (Butterfield and Najjar, 2024; Qian et al., 2024), particularly in lung cancer, where CD8^+^ T cells play a pivotal role as effectors of antitumor immunity, and their abundance and functional activity directly correlate with therapeutic efficacy (Ribas and Wolchok, 2018). However, the limited infiltration of CD8^+^ T cells into the TME, coupled with their susceptibility to exhaustion under chronic antigen exposure and inflammatory stimulation, significantly restricts their cytotoxic potential, as evidenced by impaired proliferation, reduced effector function, and upregulated inhibitory receptor (Wherry and Kurachi, 2015). To overcome T cell exhaustion, strategies such as chimeric antigen receptor T-cell therapy (Wang et al., 2024c), T-cell receptor-engineered T cells, and immune checkpoint blockade have been extensively studied. Among these, antibody-based immune checkpoint inhibitors, particularly those targeting PD-1/PD-L1 and cytotoxic T-lymphocyte-associated protein 4, have achieved durable clinical responses in multiple tumor types (Kiaie et al., 2021). Despite their success, antibody-based ICIs suffer from limitations, including high manufacturing costs, immunogenicity, poor tumor penetration, and batch-to-batch variability (Tang et al., 2021), which has driven increasing attention toward peptide-based checkpoint inhibitors. Peptides offer several advantages, including low immunogenicity, ease of synthesis, tunable structures, and enhanced tissue permeability. DPPA is a selective PD-L1 blocking peptide, which has shown great potential in immunotherapy (Wu et al., 2025). However, their clinical translation is hindered by rapid enzymatic degradation, low binding affinity, and short circulation half-lives. To address these drawbacks, nanocarrier-based delivery systems have been developed (Liu et al., 2021; Fu et al., 2022). Nevertheless, reliance solely on T cell activation remains insufficient because of the immunosuppressive tumor microenvironment. Tumor-associated macrophages (TAMs) which usually present as M2 phenotype, are abundant in immunosuppressive tumor microenvironment, exacerbating immune suppression by promoting tumor angiogenesis, extracellular matrix remodeling, metastasis, and immune evasion (Mantovani et al., 2017). Reprogramming TAMs toward an M1-like anti-tumor phenotype is therefore a promising strategy to enhance the efficacy of immunotherapy. Current approaches employing toll-like receptor (TLR) agonists (Kawai et al., 2024), CD40 agonists (Liu et al., 2023), and PI3Kγ inhibitors (Zhang et al., 2023b) have shown immunomodulatory potential. Resiquimod (R848), a typical TLR agonist, has showed validity in promoting TAMs repolarization and DCs mature by activating MyD88-dependent NF-κB and p38 MAPK signaling pathways (Bhagchandani et al., 2021). For further improving the efficiency of immunotherapy, studies have focused on multimodal therapy targeting CTLs, TAMs or DCs in immunosuppressive TME (Ding et al., 2025; Shen et al., 2026; Von Locquenghien et al., 2025).

PD-L1 antagonistic peptide DPPA has demonstrated efficiency in blocking the interaction of PD-1/PD-L1 acting as an immune checkpoint inhibitor with low cost and good stability. DPPA usually bound on the surface of nanoparticles by forming covalent linkage or phospholipid modification (Li et al., 2024; Wang et al., 2024d). In this study, we developed an acid-responsive liposome DRPL for codelivery of DPPA and Toll-like receptor agonists R848, functionalized with DSPE-PEOz. DPPA and R848 were encapsulated in the aqueous phase and phospholipid bilayer of DRPL liposome respectively, according to thin-film evaporation which is a commonly used method. DRPL showed a typical tyndall effect and morphology of liposome, with low protein adsorption and hemolysis. Tumor blood vessels exhibit abnormal architecture, characterized by discontinuous and loosely aligned endothelial cells, resulting in markedly increased vascular permeability. Concurrently, intratumoral lymphatic drainage is impaired and blood flow velocity is significantly reduced. These pathophysiological features enable the passive accumulation and prolonged retention of nanoparticles (20–200 nm) within tumor tissue, which called the enhanced permeability and retention (EPR) effect (Sun et al., 2022). DRPL reduced serum proteins attach, evading opsonization and rapid clearance by the mononuclear phagocyte system. Consequently, DRPL demonstrated extended circulation life time in vivo, fulfilling prerequisite for tumor targeting. Upon reaching the tumor via the bloodstream, DRPL crossed the hyperpermeable blood vessels into tumor stromal and retained for a long time because of the deficient lymphatic drainage, thereby achieving passive targeting. In the acidic TME (pH 6.4–6.8), oxazoline of DSPE-PEOz protonated, resulting in solubility and conformation changes, thereby leading to liposomes structural disassembly and drug release. Extracellularly released DPPA bound to PD-L1 on LLC and blocked PD-1/PD-L1 axis, thereby activating CTLs and restoring their cytotoxicity to cancer cells. On the other hand, free R848 activated toll-like receptors in TAMs and DCs, repolarizing TAMs toward pro-inflammatory M1 phenotype and promoting DCs mature. Repolarized TAMs induced LLC apoptosis and phagocytized LLC. Antigens released by LLC killed by repolarized TAMs and activated CTLs further stimulated DCs maturation and antigen presentation to CTLs, forming a valid positive immunity cycle. With remodeling of the immunosuppressive tumor microenvironment to an immunoactivated state, DRPL achieved an impressive primary tumor inhibition rate of 90.59% with minimal organ toxicity. Furthermore, DRPL demonstrated sustained immune memory response to reduce recurrence tumor weight by 82.22%. It is demonstrated a great potential of DRPL to become a powerful tool for lung cancer therapy.

## 5 Conclusion

In summary, we presented an acid-responsive liposomal immunoactivated platform named DRPL engineered for the co-delivery of PD-L1 antagonistic peptide DPPA and TLR7/8 agonist R848. DRPL demonstrated preferable stability under physiological condition and achieved efficient passive tumor accumulation. In the weakly acidic tumor microenvironment, DRPL exhibited pH-triggered structural disassembly, enabling synchronous drug release. DRPL concurrently activated CD8^+^ T cells, repolarizing tumor-associated macrophages toward an immunostimulatory phenotype and induced dendritic cell maturation. This multimodal immunomodulation translated into effective inhibition of non-small cell lung cancer growth and induced sustained immune response against tumor recurrence. This liposomal platform with versatile payload capacity accommodates both peptide therapeutics and small-molecule drugs, has translational potential to overcome immune resistance and improve immunotherapeutic efficacy in cancer treatment.

## CRediT authorship contribution statement

**Yinshan Lin:** Software, Methodology, Investigation, Formal analysis, Data curation. **Lu Liang:** Writing – review & editing, Investigation, Funding acquisition, Formal analysis, Data curation. **Le Wang:** Writing – review & editing, Supervision, Resources, Project administration. **Minyan Wei:** Writing – original draft, Formal analysis, Data curation. **Qingjun Yang:** Software, Formal analysis, Data curation. **Xiaoling Guan:** Validation, Formal analysis. **Qiuyun Liu:** Validation, Software. **Youguang Pan:** Writing – original draft, Formal analysis, Data curation. **Jianfen Su:** Writing – review & editing, Supervision, Resources, Funding acquisition. **Lingmin Zhang:** Writing – review & editing, Supervision, Project administration, Funding acquisition, Conceptualization.

## Declaration of competing interest

The authors declare that they have no known competing financial interests or personal relationships that could have appeared to influence the work reported in this paper.

## Data Availability

The authors confirm that the data supporting the findings of this study are available within the article and its supplementary materials.
