# Supplementary material for: Acidic-responsive nano liposomes potentiate in situ dual immunotherapy on lung cancer by codelivering antagonistic peptide and agonist
Source: Int J Pharm X. 2026 May 6;11:100562. doi: 10.1016/j.ijpx.2026.100562 (PMC13187613; doi:10.1016/j.ijpx.2026.100562)
Supplement: Supplementary file 1 — Supplementary material [file mmc1.docx]

**Supplementary information**

**Acidic-responsive nano liposomes potentiate in situ dual immunotherapy on lung cancer by codelivering antagonistic peptide and agonist**

Yinshan Lin ^a, 1^, Lu Liang ^a, 1^, Le Wang ^b, 1^, Minyan Wei ^a^, Qingjun Yang ^a^, Xiaoling Guan ^a^, Qiuyun Liu ^a^, Youguang Pan ^a,^ *, Jianfen Su ^a,^ *, Lingmin Zhang ^a,^ *

^a^ *The Affiliated Panyu Central Hospital, Guangzhou Municipal and Guangdong Provincial Key Laboratory of Molecular Target & Clinical Pharmacology, the NMPA and State Key Laboratory of Respiratory Disease, Guangdong Basic Research Center of Excellence for Respiratory Medicine, School of Pharmaceutical Sciences, Guangzhou Medical University, Guangzhou, 511436, China*

^b^ *Academician Workstation, Department of Pharmacy, The Jiangxi Province Key Laboratory for Diagnosis, Treatment, and Rehabilitation of cancer in Chinese Medicine, Jiangxi University of Chinese Medicine, No. 1688 Meiling Avenue, Xinjian District, Nanchang, Jiangxi, 330004, China.*

*Corresponding authors.

E-mail address: zhanglm@gzhmu.edu.cn (L. Zhang), sujianfen@pyhospital.com.cn (J. Su), and panyouguang@126.com (Y. Pan)

^1^ These authors contributed to this work equally.

**Table. S1.** Sequence of Primers

| Gene | Sequence | Product length |
| --- | --- | --- |
| Arg1 | F: CATTGGCTTGCGAGACGTAGAC  R: GCTGAAGGTCTCTTCCATCACC | 124 |
| IL10 | F: CAAAGGACCAGCTGGACAACA  R: GCAACCCAAGTAACCCTTAAAGTC | 71 |
| TGFβ | F: CAACAATTCCTGGCGTTACCTTGG  R: GAAAGCCCTGTATTCCGTCTCCTT | 128 |
| iNOS | F: TGACGCTCGGAACTGTAGCAC  R: TGATGGCCGACCTGATGTTGC | 98 |
| CD80 | F: GCAATTGTCAGTTGATGCAGGA  R: AGTTGTAACGGCAAGGCAGC | 165 |
| TNFα | F: CCCTCACACTCAGATCATCTTCT  R: GCTACGACGTGGGCTACAG | 61 |
| CD86 | F: AGTTGGTTCTGTACGAGCACTA  R: CATTGTGAAGTCGTAGAGTCCAG | 114 |
| CCR7 | F: GCAACGGGCTGGTGATACTG  R: ACTTGGCTTCGCTGTAGGC | 138 |
| IL1β | F: CTTCAGGCAGGCAGTATCACTCAT  R: TCTAATGGGAACGTCACACACCAG | 187 |
| IL6 | F: TTCCATCCAGTTGCCTTCTTG  R: TTGGGAGTGGTATCCTCTGTGA | 101 |
| IL12 | F: GCATGTGTCAATCACGCTACCT  R: ACCATGTCATCTGTGGTCTTCAG | 145 |
| GAPDH | F: TGCACCACCAACTGCTTAGC  R: GGCATGGACTGTGGTCATGAG | 87 |


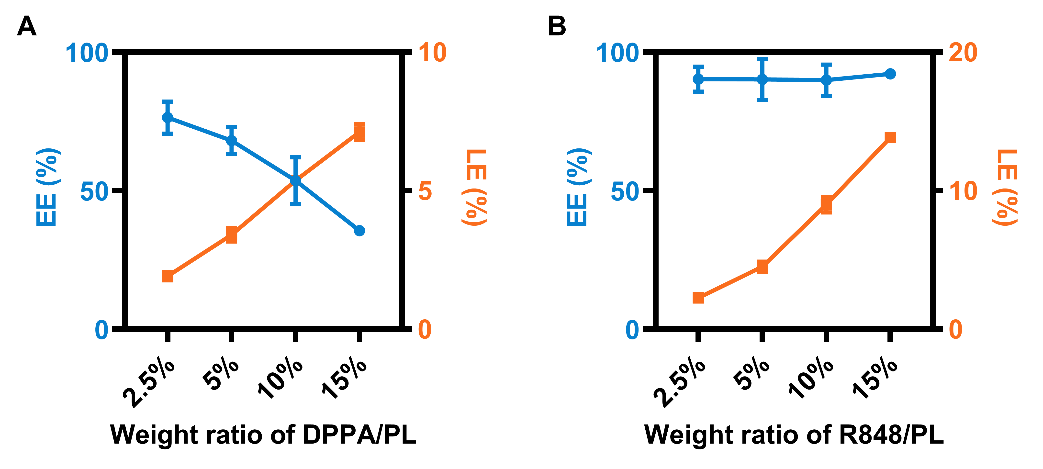


**Fig. S1.** The encapsulation efficiency and loading efficiency of different cargos by the liposomes. (A) DPPA. (B) R848. EE, encapsulation efficiency. LE, loading efficiency. Data are represented as mean ± SD, *n* = 3.


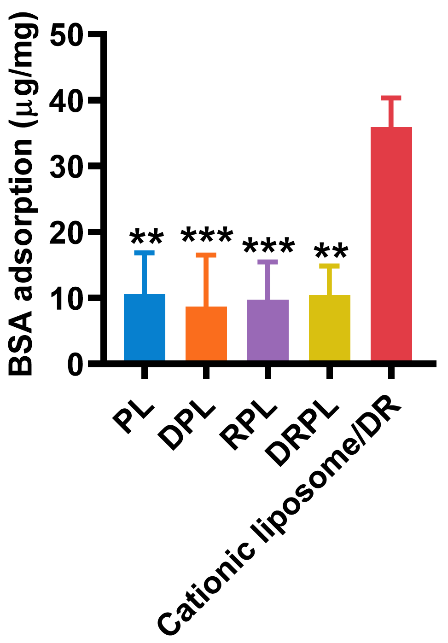


**Fig. S2.** Protein affinity of different formulations. PL, PEOz-liposome; DPL, DPPA @PEOz-liposome; RPL, R848@PEOz-liposome; DRPL, DPPA+R848@PEOz-liposome; Cationic liposome/DR, DPPA+R848@Cationic liposome. Data are represented as mean ± SD, *n* = 3; ** *P* < 0.01, and ****P* < 0.001 vs Cationic liposome/DR.


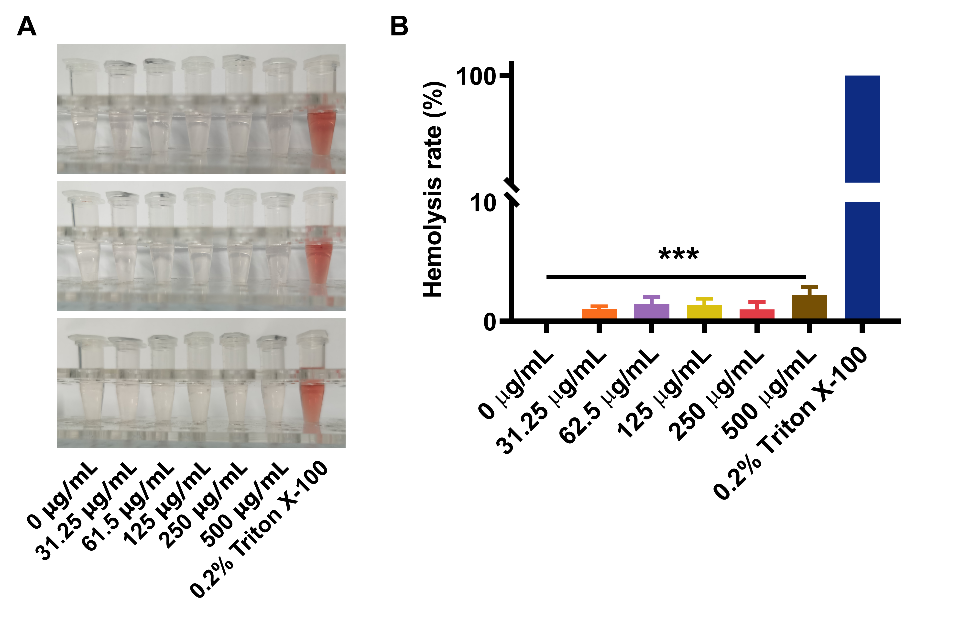


**Fi****g. S3.** Hemolysis of DRPL. (A) Images of DRPL in different concentration incubated with 2% RBSs; (B) Hemolysis rate of DRPL in different concentration. Data are represented as mean ± SD, *n* = 3; **P* < 0.05, ***P* < 0.01, and ****P* < 0.001 vs 0.2% Triton X-100.


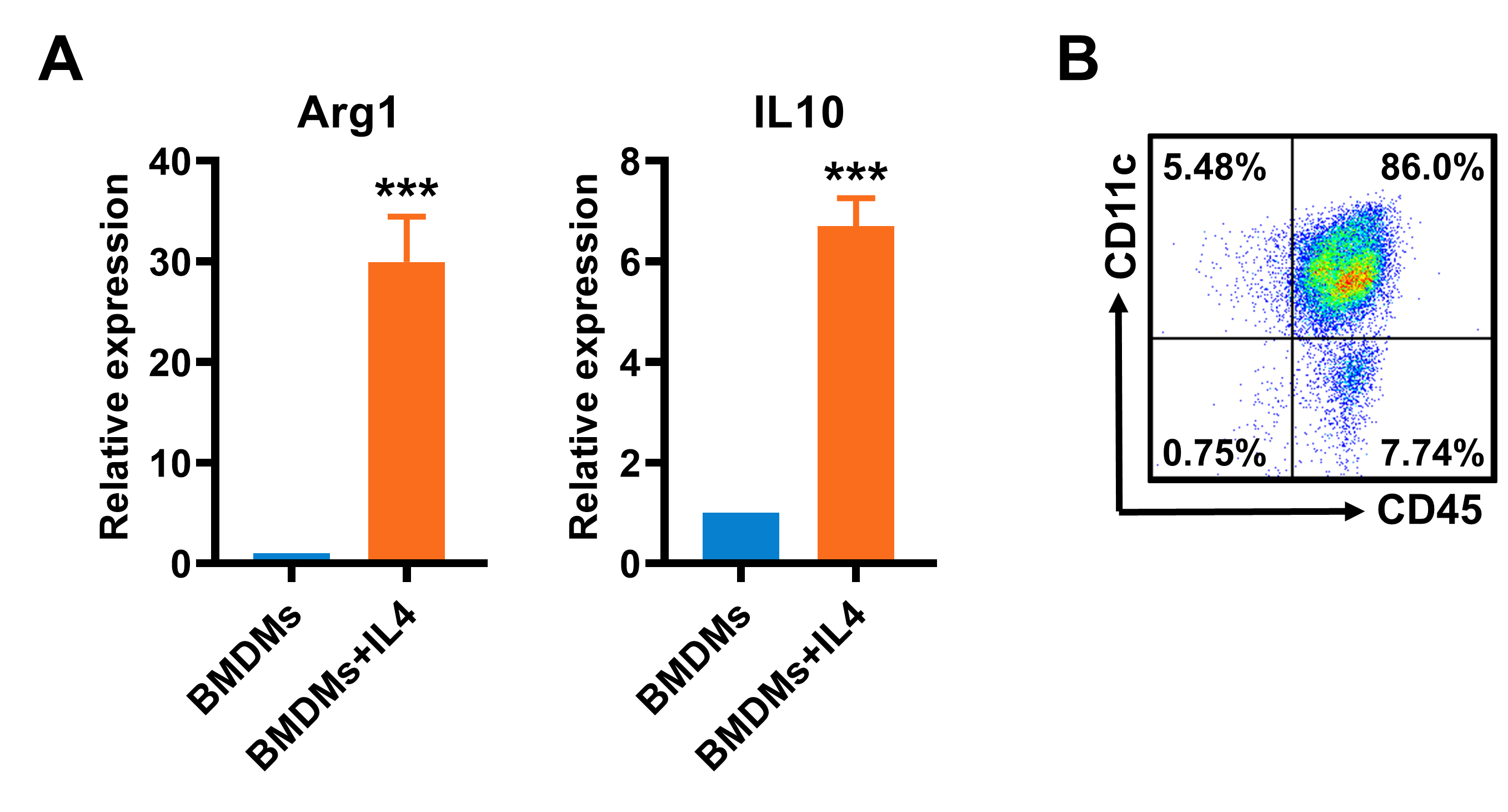


**Fig. S4.** Characterization of BMDMs and BMDCs. (A) Relative Arg1 and IL10 mRNA expression of BMDMs and BMDMs+IL4. Data are represented as mean ± SD, *n* = 3, ****P* < 0.001; (B) FCAS analysis of CD45^+^CD11c^+^ BMDCs.

**
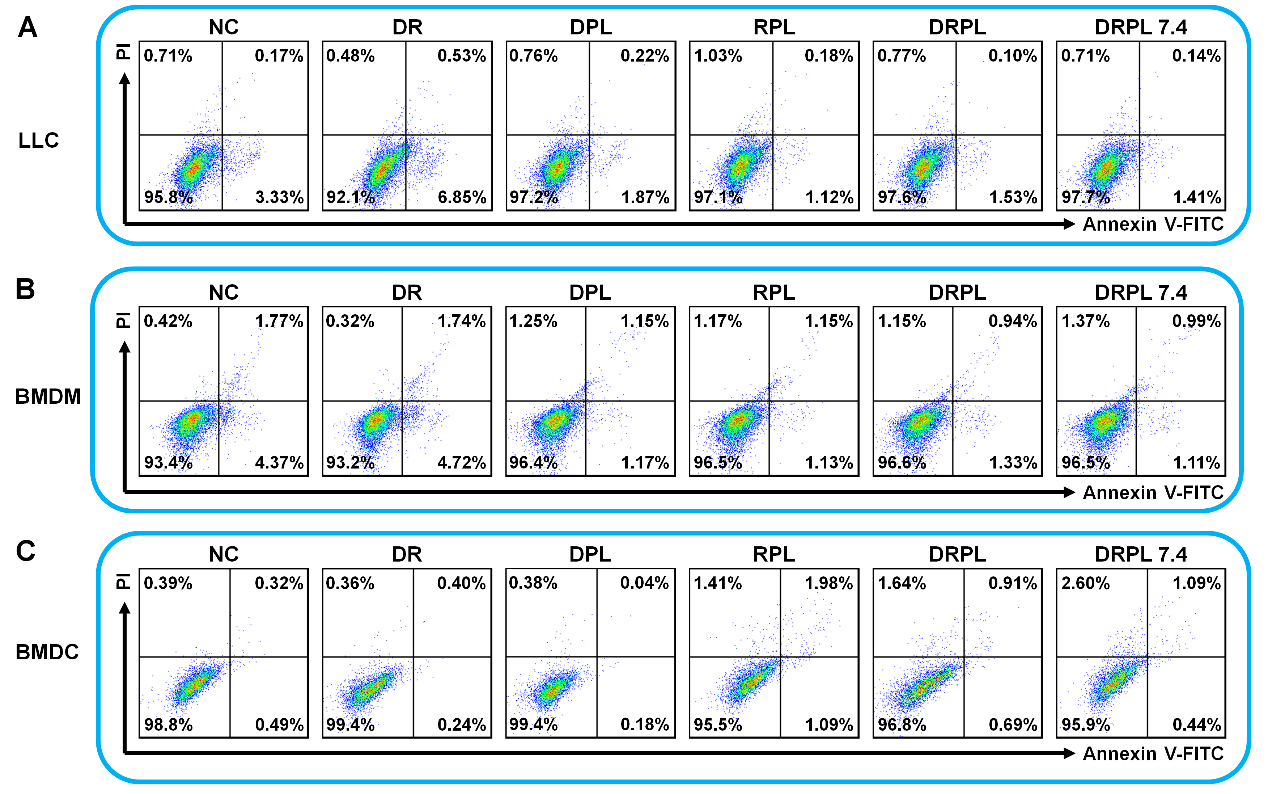
**

**Fig. S5.** Cells apoptosis induced by different formulations. (A) LLC, (B) BMDMs and (C) BMDCs were treated with NC, DR, DPL, RPL, DRPL and DRPL 7.4 for 24 h in pH 6.4. NC, PBS; DR, DPPA+R848; DPL, DPPA @PEOz-liposome; RPL, R848@PEOz-liposome; DRPL, DPPA+R848@PEOz-liposome.


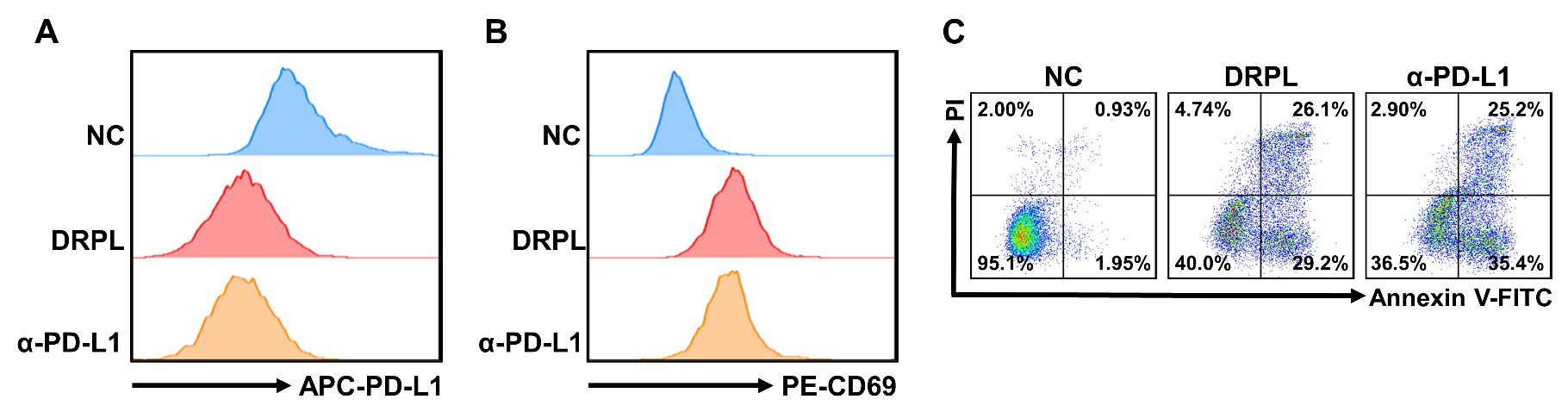


**Fig. S6.** Comparison of DRPL and α-PD-L1 in pH 6.5 culture medium. (A) PD-L1 blockade of LLC after treated with DRPL or α-PD-L1 in pH 6.5 culture medium. (B) T cells activation after coculturing with LLC pretreated with DRPL or α-PD-L1 in pH 6.5 culture medium. (C) Apoptosis of LLC pretreated with DRPL or α-PD-L1 in pH 6.5 culture medium after coculturing with T cells for 24 h.


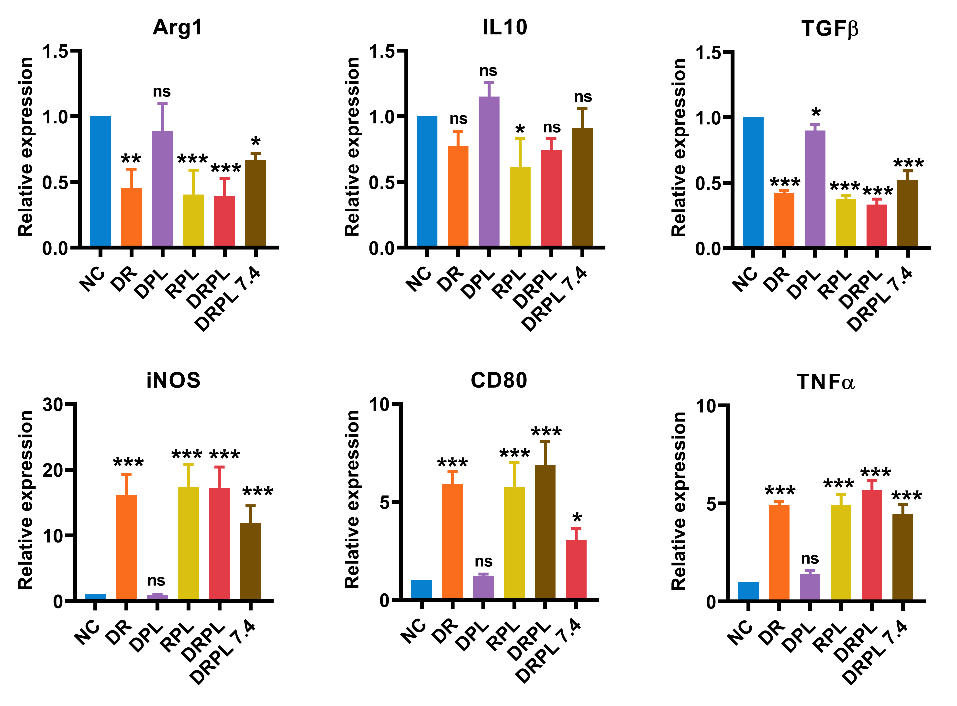


**Fig. S7.** Relative mRNA level of Arg1, IL10, TGFβ, iNOS, CD80, and TNFα of BMDMs after treatment with PBS, DR, DPL, RPL, DRPL, and DRPL 7.4 for 24 h. NC, PBS; DR, DPPA+R848; DPL, DPPA@PEOz-liposome; RPL, R848@PEOz-liposome; DRPL, DPPA+R848@PEOz-liposome. Data are represented as mean ± SD, *n* = 3; **P* < 0.05, ***P* < 0.01, and ****P* < 0.001 vs NC. ns, no significant differences.


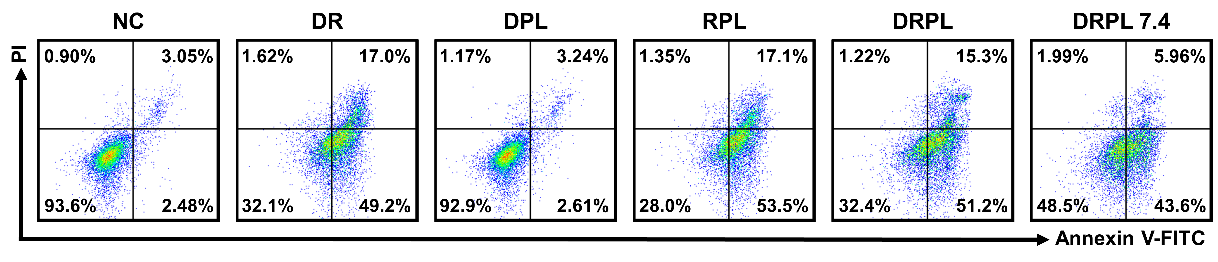


**Fig. S8.** Cell apoptosis of LLC after coculture with BMDMs pretreated with different formulations for 48 h. NC, PBS; DR, DPPA+R848; DPL, DPPA @PEOz-liposome; RPL, R848@PEOz-liposome; DRPL, DPPA+R848@PEOz-liposome.

**Fig. S9.** Phagocytosis of BMDMs pretreated with different formulations to GFP-LLC after coculture for 4 h. NC, PBS; DR, DPPA+R848; DPL, DPPA @PEOz-liposome; RPL, R848@PEOz-liposome; DRPL, DPPA+R848@PEOz-liposome. Data are represented as mean ± SD, *n* = 3; **P* < 0.05, ***P* < 0.01, and ****P* < 0.001 vs NC. ns, no significant differences.

**Fig. S10.** Colonies number of LLC after coculture with BMDMs pretreated with different formulations for 7 days. NC, PBS; DR, DPPA+R848; DPL, DPPA @PEOz-liposome; RPL, R848@PEOz-liposome; DRPL, DPPA+R848@PEOz-liposome. Data are represented as mean ± SD, *n* = 3; **P* < 0.05, ***P* < 0.01, and ****P* < 0.001 vs NC. ns, no significant differences.


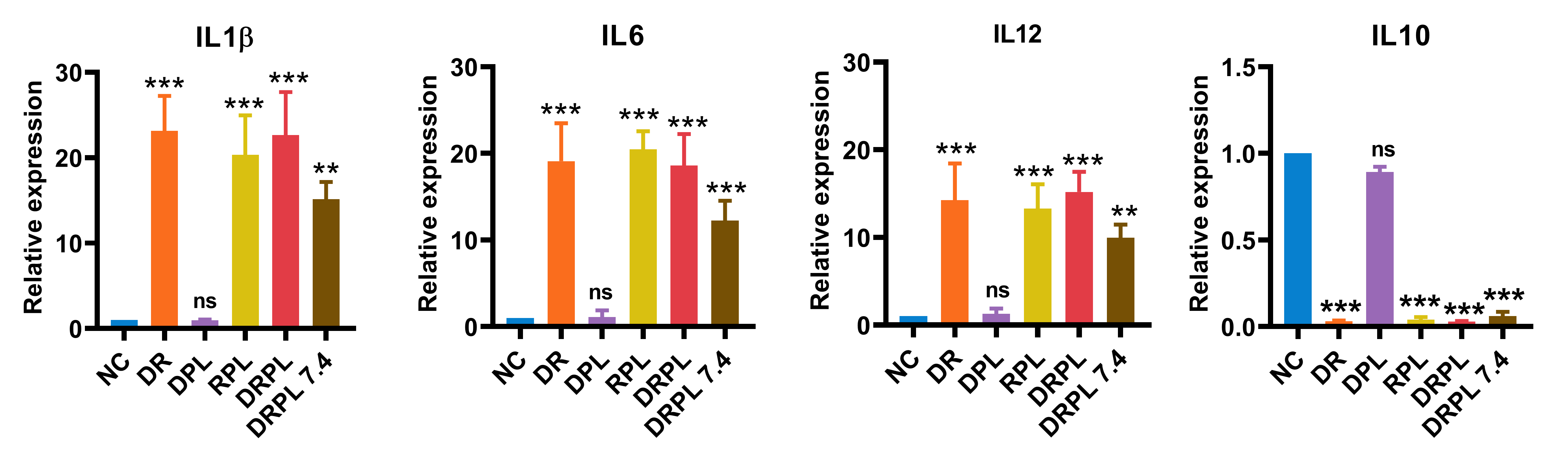


**Fig. S11.** Relative mRNA levels of IL1β, IL6, IL12 and IL10 in BMDCs after treatment with PBS, DR, DPL, RPL, DRPL, and DRPL 7.4. NC, PBS; DR, DPPA+R848; DPL, DPPA @PEOz-liposome; RPL, R848@PEOz-liposome; DRPL, DPPA+R848@PEOz-liposome. Data are represented as mean ± SD, *n* = 3; **P* < 0.05, ***P* < 0.01, and ****P* < 0.001 vs NC. ns, no significant differences.

**Fig. S12.** Fluorescence intensity of DRPL in blood within 48 h. Data are represented as mean ± SD, *n* = 3.

**Fig. S13.** Fluorescence intensity of tumors and major organs at 48 h post-injection. Data are represented as mean ± SD, *n* = 3; **P* < 0.05, ***P* < 0.01, and ****P* < 0.001 vs DRPL. ns, no significant differences.

**
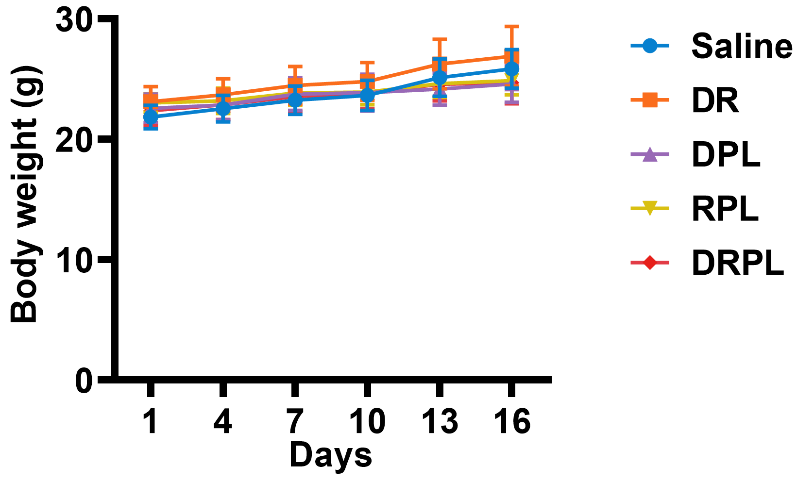
**

**Fig. S14.** Body weight changes during treatment. DR, DPPA+R848; DPL, DPPA @PEOz-liposome; RPL, R848@PEOz-liposome; DRPL, DPPA+R848@PEOz-liposome. Data are represented as mean ± SD, *n* = 6.


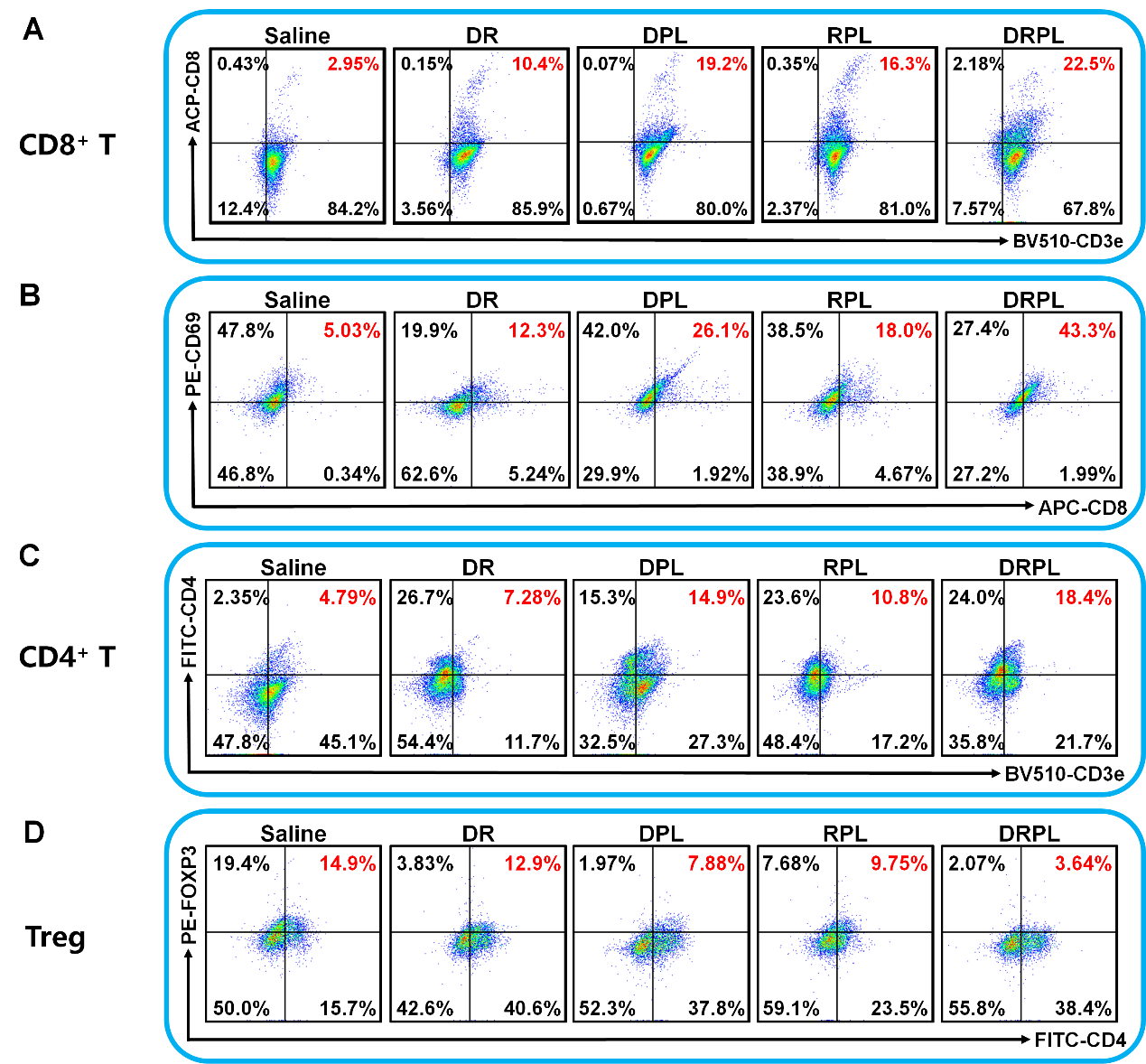


**Fig. S15.** Flow cytometry analysis of T cells in tumors after different treatment. (A) CD8^+^ T cells; (B) Activated CD8^+^ T cells; (C) CD4^+^ T cells; (D) Treg cells in CD45^+^ cells from tumors. DR, DPPA+R848; DPL, DPPA @PEOz-liposome; RPL, R848@PEOz-liposome; DRPL, DPPA+R848@PEOz-liposome.


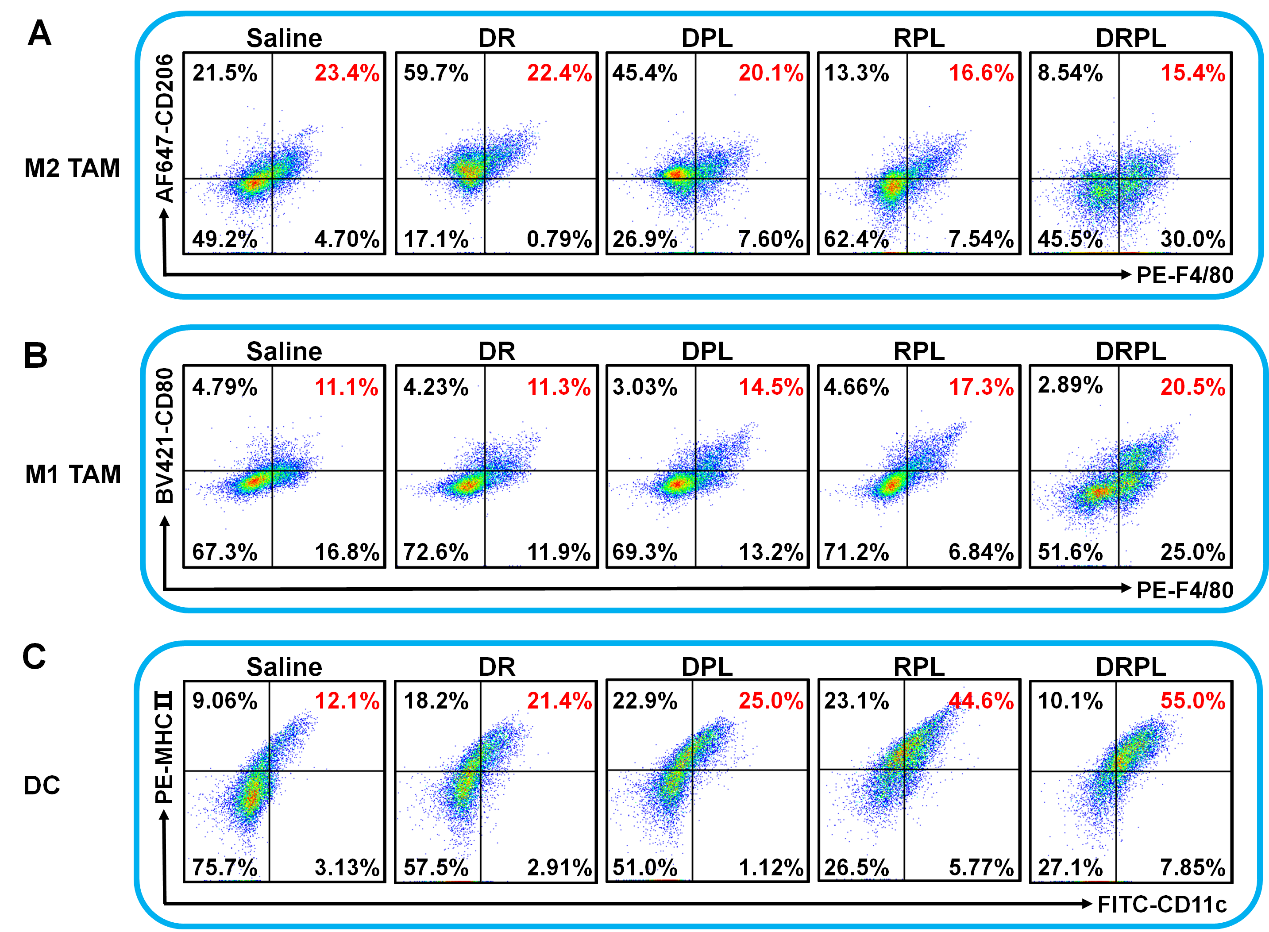


**Fig. S16.** Flow cytometry analysis of tumor associated macrophages and dendritic cells in tumors after different treatment. (A) M2 TAM; (B) M1 TAM; (C) Dendritic cells in CD45^+^ cells from tumors. DR, DPPA+R848; DPL, DPPA @PEOz-liposome; RPL, R848@PEOz-liposome; DRPL, DPPA+R848@PEOz-liposome.

**Fig. S17.** Flow cytometry analysis of CD4^+^ T cells in spleens after different treatment. DR, DPPA+R848; DPL, DPPA @PEOz-liposome; RPL, R848@PEOz-liposome; DRPL, DPPA+R848@PEOz-liposome. Data are represented as mean ± SD, *n* = 5; **P* < 0.05, ***P* < 0.01, and ****P* < 0.001 vs. DRPL.


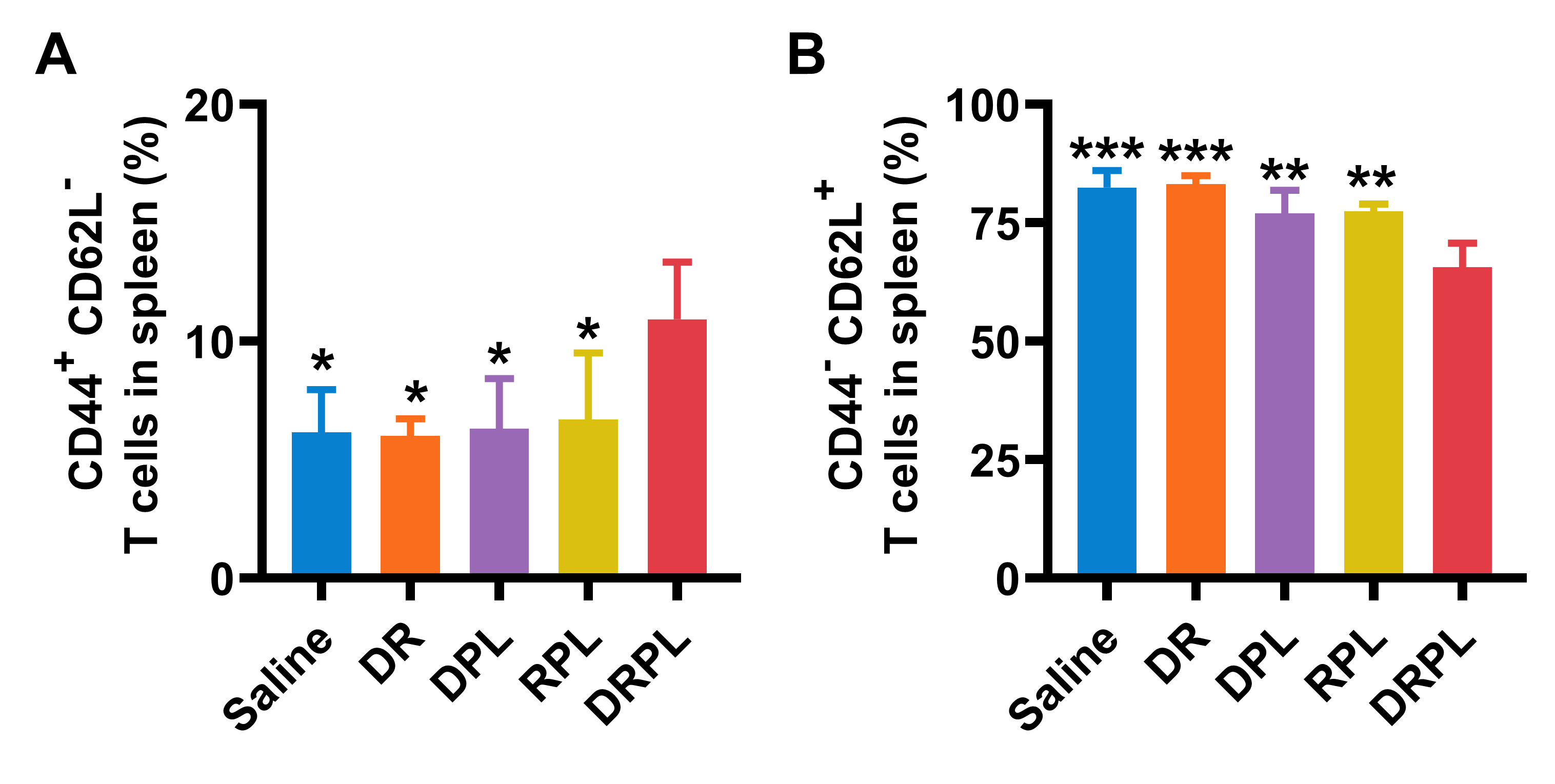


**Fig. S18.** Flow cytometry analysis of T cells status in spleens after different treatment. (A) Effector T cells and (B) Naive T cells in CD45^+^ CD3e^+^ cells from spleens. DR, DPPA+R848; DPL, DPPA@PEOz-liposome; RPL, R848@PEOz-liposome; DRPL, DPPA+R848@PEOz-liposome. Data are represented as mean ± SD, *n* = 5; **P* < 0.05, ***P* < 0.01, and ****P* < 0.001 vs. DRPL.
